# 2,4-Di-*tert*-butyl-6-[(*R*/*S*)-1-(3,5-di-*tert*-butyl-2-hy­droxy­phen­yl)eth­yl]phenyl [(1*R*,4*S*)-7,7-dimethyl-2-oxobicyclo­[2.2.1]heptan-1-yl]methane­sulfonate

**DOI:** 10.1107/S1600536811052664

**Published:** 2011-12-10

**Authors:** Cheng Wang, Jincai Wu

**Affiliations:** a Department of Thoracis Surgery, The Second Hospital of Lanzhou University, Lanzhou 730000, People’s Republic of China; b Department of Chemistry, Lanzhou University, Lanzhou 730000, People’s Republic of China

## Abstract

The asymmetric unit of the title compound, C_40_H_60_O_5_S, comprises two diastereomers related, except for the chiral camphor groups, by a pseudo-inversion centre. In both diasteromers, the camphor sulfate moiety maintained the absolute configuartion (*R*,*S*) of the precursor. However, the absolute configurations at the methine C atoms are of opposite chirality. Both mol­ecules reveal intra­molecular O—H⋯O hydrogen bonds, whereas van der Waals inter­actions define the crystal packing.

## Related literature

The title compound is a potential ligand for the investigation of ring-opening polymerization of lactides. Poly(lactide) and poly(∊-caprolactone) and their copolymers are the most promising biodegradable and biocompatible synthetic macromolecules. Due to the advantages of well controlled mol­ecular weight and low polydispersity, many metal complexes have been used, see: Wu *et al.* (2006 ▶).
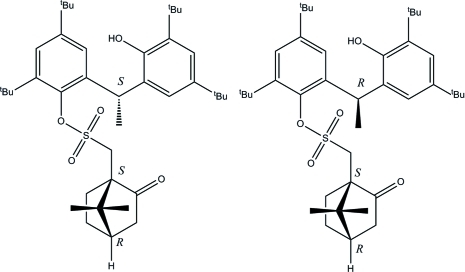


## Experimental

### 

#### Crystal data

C_40_H_60_O_5_S
*M* *_r_* = 652.98
Monoclinic, 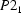

*a* = 18.2082 (19) Å
*b* = 10.6028 (11) Å
*c* = 20.287 (2) Å
β = 93.003 (2)°
*V* = 3911.2 (7) Å^3^
*Z* = 4
Mo *K*α radiation
μ = 0.12 mm^−1^
*T* = 293 K
0.32 × 0.28 × 0.23 mm

#### Data collection

Bruker SMART CCD area-detector diffractometer
Absorption correction: multi-scan (*SADABS*; Bruker, 1998) ▶ *T*_min_ = 1, *T*_max_ = 1
21496 measured reflections
13182 independent reflections
7458 reflections with *I* > 2σ(*I*)
*R*_int_ = 0.045

#### Refinement

*R*[*F* ^2^ > 2σ(*F* ^2^)] = 0.069
*wR*(*F* ^2^) = 0.203
*S* = 1.08
13182 reflections
829 parameters
53 restraints
H-atom parameters constrained
Δρ_max_ = 0.88 e Å^−3^
Δρ_min_ = −0.35 e Å^−3^
Absolute structure: Flack (1983 ▶), 5058 Friedel pairs
Flack parameter: −0.12 (12)

### 

Data collection: *SMART* (Bruker, 1998 ▶); cell refinement: *SAINT-Plus* (Bruker, 1998 ▶); data reduction: *SAINT-Plus*; program(s) used to solve structure: *SHELXS97* (Sheldrick, 2008 ▶); program(s) used to refine structure: *SHELXL97* (Sheldrick, 2008 ▶); molecular graphics: *SHELXTL* (Sheldrick, 2008 ▶); software used to prepare material for publication: *SHELXTL* and *PLATON* (Spek, 2009 ▶).

## Supplementary Material

Crystal structure: contains datablock(s) I, global. DOI: 10.1107/S1600536811052664/kp2372sup1.cif

Structure factors: contains datablock(s) I. DOI: 10.1107/S1600536811052664/kp2372Isup2.hkl

Supplementary material file. DOI: 10.1107/S1600536811052664/kp2372Isup3.cml

Additional supplementary materials:  crystallographic information; 3D view; checkCIF report

## Figures and Tables

**Table 1:** Hydrogen-bond geometry (Å, °)

*D*—H⋯*A*	*D*—H	H⋯*A*	*D*⋯*A*	*D*—H⋯*A*
O1—H1*A*⋯O3	0.82	2.23	2.974 (6)	152
O1*A*—H1*AA*⋯O3*A*	0.82	2.20	2.957 (6)	153

**Fig. 1.**
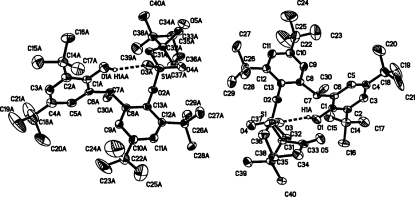
The structure of (I) with the two molecules in an asymmetric unit showing displacement ellipsoids drawn at the 30% probability level. Intramolecular hydrogen bonds are shown (in dashed lines).

## supplementary crystallographic information

### Comment

Poly(lactide) and poly(ε-caprolactone) and their copolymers are the most promising biodegradable and biocompatible synthetic macromolecules. Due to the advantages of well controlled molecular weight and low polydispersity (PDI), many metal complexes have been used (Wu *et al.*, 2006). In the present study, we report a structure which is a potential ligand for the investigation of ring-opening polymerisation of lactides. Camphor sulfate moiety mentains *R*,*S* configuration and the chiral centre at methine  atoms C7 and C7A are of opposite chirality. Thus, there are two diastereomers in one unsymmetrical unit with the absolute configurations (*R*, *R*, *S*) and (*S*, *R*, *S*) respectively (Scheme and Fig. 1). The bond lengths and angles are within normal range. There are two intramolecular hydrogen bonds of O2—H1a—O3 and O2a—H1aa—O3a (Table 1 and Fig. 1).

### Experimental

6,6'-(ethane-1,1-diyl)bis(2,4-di-*tert*-butylphenol)(4.38 g, 10 mmol) and triethylamine (14 mL, 100 mmol) were dissolved in 100 mL of dichloromethane. (*R*,*S*)camphor sulfonyl chloride (2.76 g, 11 mmol) in dichloromethane (20 mL) was added dropwise into the above solution at 273 K for about 1 h. Then, the resulting mixture was stirred for 24 h while the temperature was increased to room temperature. The solution was filtered, and the filtrate was washed with 50 mL of water three times. The dichloromethane layer was collected and dried over anhydrous MgSO_4_ and filtered through Celite again to remove MgSO_4_. The resulting filtrate was then dried under vacuo, and the residue was recrystallised by slow cooling of a acetonitrile solution.

### Refinement

*PLATON*/ADDSYM (Spek, 2009) suggested an inversion symmetry. However, this was rejected, since the two molecules in one asymmetric unit are diastereomers and related by pseudo-inversion centre. Due to disorders of *tert*-butyl groups, 53 restrains were applied.

The C-bound H atoms were placed at calculated positions and were treated as riding on their parent C atoms with C—H= 0.95 Å. The O-bound H atom was located in a difference Fourier map, and refined with distance restraints of O—H = 0.84 (2) Å.

### Crystal data

**Table:**

C_40_H_60_O_5_S	*F*(000) = 1424
*M**_r_* = 652.98	*D*_x_ = 1.109 Mg m^−^^3^
Monoclinic, *P*2_1_	Mo *K*α radiation, λ = 0.71073 Å
Hall symbol: P 2yb	Cell parameters from 5452 reflections
*a* = 18.2082 (19) Å	θ = 2.2–24.4°
*b* = 10.6028 (11) Å	µ = 0.12 mm^−^^1^
*c* = 20.287 (2) Å	*T* = 293 K
β = 93.003 (2)°	Block, colorless
*V* = 3911.2 (7) Å^3^	0.32 × 0.28 × 0.23 mm
*Z* = 4	

### Data collection

**Table:**

Bruker SMART CCD area-detector diffractometer	13182 independent reflections
Radiation source: fine-focus sealed tube	7458 reflections with *I* > 2σ(*I*)
graphite	*R*_int_ = 0.045
phi and ω scans	θ_max_ = 26.1°, θ_min_ = 2.0°
Absorption correction: multi-scan (*SADABS*; Bruker, 1998)	*h* = −22→22
*T*_min_ = 1, *T*_max_ = 1	*k* = −13→12
21496 measured reflections	*l* = −21→25

### Refinement

**Table:**

Refinement on *F*^2^	Secondary atom site location: difference Fourier map
Least-squares matrix: full	Hydrogen site location: inferred from neighbouring sites
*R*[*F*^2^ > 2σ(*F*^2^)] = 0.069	H-atom parameters constrained
*wR*(*F*^2^) = 0.203	*w* = 1/[σ^2^(*F*_o_^2^) + (0.1*P*)^2^ + 0.020*P*] where *P* = (*F*_o_^2^ + 2*F*_c_^2^)/3
*S* = 1.08	(Δ/σ)_max_ = 0.001
13182 reflections	Δρ_max_ = 0.88 e Å^−^^3^
829 parameters	Δρ_min_ = −0.35 e Å^−^^3^
53 restraints	Absolute structure: Flack (1983), 5058 Friedel pairs
Primary atom site location: structure-invariant direct methods	Flack parameter: −0.12 (12)

### Special details

**Table:**

Geometry. All e.s.d.'s (except the e.s.d. in the dihedral angle between two l.s. planes) are estimated using the full covariance matrix. The cell e.s.d.'s are taken into account individually in the estimation of e.s.d.'s in distances, angles and torsion angles; correlations between e.s.d.'s in cell parameters are only used when they are defined by crystal symmetry. An approximate (isotropic) treatment of cell e.s.d.'s is used for estimating e.s.d.'s involving l.s. planes.
Refinement. Refinement of *F*^2^ against ALL reflections. The weighted *R*-factor *wR* and goodness of fit *S* are based on *F*^2^, conventional *R*-factors *R* are based on *F*, with *F* set to zero for negative *F*^2^. The threshold expression of *F*^2^ > σ(*F*^2^) is used only for calculating *R*-factors(gt) *etc*. and is not relevant to the choice of reflections for refinement. *R*-factors based on *F*^2^ are statistically about twice as large as those based on *F*, and *R*- factors based on ALL data will be even larger.

### Fractional atomic coordinates and isotropic or equivalent isotropic displacement parameters (Å^2^)

**Table:**

	*x*	*y*	*z*	*U*_iso_*/*U*_eq_	
S1	0.58400 (7)	0.22591 (15)	0.10873 (6)	0.0523 (4)	
S1A	0.41355 (7)	0.73098 (15)	0.38216 (6)	0.0514 (4)	
O1	0.76708 (19)	0.0372 (4)	0.04685 (18)	0.0652 (11)	
H1A	0.7340	0.0696	0.0670	0.098*	
O1A	0.2269 (2)	0.9138 (4)	0.44606 (19)	0.0638 (11)	
H1AA	0.2621	0.8845	0.4275	0.096*	
O2	0.58858 (16)	0.1566 (4)	0.17932 (16)	0.0459 (9)	
O2A	0.41002 (17)	0.8119 (4)	0.31519 (15)	0.0467 (9)	
O3	0.65558 (17)	0.2260 (4)	0.08357 (18)	0.0631 (10)	
O3A	0.34352 (18)	0.7361 (5)	0.41029 (17)	0.0657 (11)	
O4	0.5475 (2)	0.3416 (4)	0.1151 (2)	0.0698 (12)	
O4A	0.4438 (2)	0.6103 (4)	0.36803 (19)	0.0666 (11)	
O5	0.4662 (2)	−0.1248 (4)	0.0597 (2)	0.0716 (11)	
O5A	0.5466 (3)	0.5991 (5)	0.4890 (2)	0.0851 (13)	
C1	0.7841 (3)	−0.0787 (5)	0.0738 (3)	0.0455 (13)	
C1A	0.2110 (3)	1.0324 (6)	0.4213 (2)	0.0443 (13)	
C2A	0.1521 (3)	1.0967 (6)	0.4489 (3)	0.0519 (15)	
C2	0.8419 (3)	−0.1448 (6)	0.0475 (2)	0.0458 (13)	
C3A	0.1357 (3)	1.2142 (6)	0.4228 (3)	0.0535 (14)	
H3AB	0.0960	1.2573	0.4392	0.064*	
C3	0.8601 (3)	−0.2618 (6)	0.0753 (3)	0.0527 (14)	
H3B	0.8977	−0.3082	0.0577	0.063*	
C4A	0.1740 (3)	1.2727 (6)	0.3741 (3)	0.0541 (16)	
C4	0.8234 (3)	−0.3128 (5)	0.1293 (3)	0.0531 (15)	
C5A	0.2333 (3)	1.2052 (6)	0.3516 (3)	0.0522 (15)	
H5AA	0.2610	1.2420	0.3195	0.063*	
C5	0.7655 (3)	−0.2452 (6)	0.1513 (2)	0.0498 (14)	
H5A	0.7389	−0.2789	0.1850	0.060*	
C6	0.7448 (3)	−0.1282 (6)	0.1255 (2)	0.0436 (13)	
C6A	0.2533 (3)	1.0865 (6)	0.3742 (2)	0.0441 (14)	
C7	0.6795 (2)	−0.0552 (5)	0.1519 (2)	0.0444 (13)	
H7A	0.6545	−0.0133	0.1140	0.053*	
C7A	0.3186 (2)	1.0163 (6)	0.3489 (2)	0.0473 (14)	
H7AA	0.3416	0.9697	0.3863	0.057*	
C8	0.7033 (3)	0.0485 (6)	0.2012 (2)	0.0448 (13)	
C8A	0.2953 (2)	0.9186 (6)	0.2953 (2)	0.0426 (13)	
C9A	0.2275 (3)	0.9298 (6)	0.2607 (2)	0.0493 (14)	
H9AA	0.1952	0.9927	0.2726	0.059*	
C9	0.7694 (3)	0.0356 (6)	0.2379 (3)	0.0543 (15)	
H9A	0.8011	−0.0292	0.2272	0.065*	
C10A	0.2070 (3)	0.8494 (6)	0.2088 (3)	0.0533 (15)	
C10	0.7897 (3)	0.1141 (6)	0.2888 (3)	0.0624 (17)	
C11A	0.2559 (3)	0.7603 (6)	0.1921 (3)	0.0538 (15)	
H11A	0.2421	0.7070	0.1573	0.065*	
C11	0.7391 (3)	0.2067 (6)	0.3058 (3)	0.0586 (16)	
H11B	0.7515	0.2588	0.3416	0.070*	
C12A	0.3261 (2)	0.7417 (5)	0.2230 (2)	0.0438 (12)	
C12	0.6708 (2)	0.2249 (6)	0.2719 (3)	0.0485 (13)	
C13A	0.3409 (3)	0.8213 (5)	0.2777 (2)	0.0427 (13)	
C13	0.6567 (3)	0.1452 (5)	0.2174 (2)	0.0440 (13)	
C14	0.8834 (3)	−0.0946 (6)	−0.0104 (3)	0.0592 (16)	
C14A	0.1073 (3)	1.0395 (6)	0.5042 (3)	0.0560 (15)	
C15A	0.0478 (3)	1.1307 (8)	0.5263 (4)	0.090 (2)	
H15A	0.0138	1.1484	0.4897	0.135*	
H15B	0.0704	1.2078	0.5416	0.135*	
H15C	0.0220	1.0929	0.5613	0.135*	
C15	0.9220 (3)	0.0290 (7)	0.0098 (3)	0.081 (2)	
H15D	0.9557	0.0139	0.0469	0.121*	
H15E	0.8860	0.0900	0.0215	0.121*	
H15F	0.9485	0.0603	−0.0265	0.121*	
C16	0.8286 (3)	−0.0746 (8)	−0.0706 (3)	0.077 (2)	
H16A	0.8054	−0.1533	−0.0823	0.116*	
H16B	0.8547	−0.0438	−0.1072	0.116*	
H16C	0.7919	−0.0143	−0.0594	0.116*	
C16A	0.1590 (3)	1.0149 (8)	0.5649 (3)	0.077 (2)	
H16D	0.1967	0.9566	0.5536	0.116*	
H16E	0.1315	0.9799	0.5996	0.116*	
H16F	0.1812	1.0928	0.5796	0.116*	
C17	0.9419 (3)	−0.1892 (7)	−0.0329 (3)	0.082 (2)	
H17A	0.9770	−0.2054	0.0031	0.123*	
H17B	0.9666	−0.1542	−0.0693	0.123*	
H17C	0.9183	−0.2667	−0.0463	0.123*	
C17A	0.0702 (3)	0.9203 (8)	0.4818 (4)	0.086 (2)	
H17D	0.0369	0.9377	0.4447	0.128*	
H17E	0.0434	0.8860	0.5171	0.128*	
H17F	0.1066	0.8605	0.4692	0.128*	
C18A	0.1533 (3)	1.4015 (7)	0.3465 (4)	0.084 (2)	
C18	0.8457 (4)	−0.4404 (6)	0.1582 (3)	0.0734 (19)	
C19	0.9206 (4)	−0.4849 (9)	0.1387 (5)	0.147 (4)	
H19A	0.9207	−0.4920	0.0915	0.221*	
H19B	0.9312	−0.5657	0.1583	0.221*	
H19C	0.9574	−0.4253	0.1538	0.221*	
C19A	0.0802 (5)	1.4329 (13)	0.3492 (7)	0.199 (7)	
H19D	0.0682	1.4432	0.3944	0.299*	
H19E	0.0504	1.3669	0.3293	0.299*	
H19F	0.0708	1.5103	0.3257	0.299*	
C20A	0.1444 (8)	1.3807 (13)	0.2671 (5)	0.229 (8)	
H20A	0.1315	1.4593	0.2460	0.344*	
H20B	0.1063	1.3199	0.2571	0.344*	
H20C	0.1900	1.3507	0.2513	0.344*	
C20	0.8493 (6)	−0.4350 (10)	0.2355 (4)	0.152 (4)	
H20D	0.8633	−0.5162	0.2530	0.228*	
H20E	0.8019	−0.4123	0.2504	0.228*	
H20F	0.8850	−0.3733	0.2505	0.228*	
C21A	0.2125 (5)	1.4869 (10)	0.3425 (7)	0.190 (7)	
H21A	0.2266	1.5178	0.3858	0.285*	
H21B	0.1977	1.5563	0.3145	0.285*	
H21C	0.2535	1.4445	0.3245	0.285*	
C21	0.7926 (7)	−0.5357 (8)	0.1300 (8)	0.237 (9)	
H21D	0.7929	−0.5340	0.0827	0.355*	
H21E	0.7441	−0.5163	0.1434	0.355*	
H21F	0.8066	−0.6181	0.1457	0.355*	
C22A	0.1319 (4)	0.8681 (8)	0.1724 (4)	0.092 (2)	
C22	0.8636 (4)	0.0962 (8)	0.3285 (4)	0.096 (2)	
C26A	0.3784 (3)	0.6413 (5)	0.1954 (3)	0.0483 (14)	
C26	0.6184 (3)	0.3277 (6)	0.2964 (3)	0.0577 (16)	
C27	0.6386 (4)	0.3601 (7)	0.3692 (3)	0.088 (2)	
H27A	0.6344	0.2858	0.3958	0.132*	
H27B	0.6056	0.4237	0.3840	0.132*	
H27C	0.6881	0.3911	0.3733	0.132*	
C27A	0.3650 (4)	0.5120 (6)	0.2267 (3)	0.0762 (19)	
H27D	0.3741	0.5175	0.2736	0.114*	
H27E	0.3975	0.4510	0.2090	0.114*	
H27F	0.3149	0.4868	0.2170	0.114*	
C28	0.5395 (3)	0.2814 (7)	0.2945 (3)	0.078 (2)	
H28A	0.5363	0.2077	0.3217	0.116*	
H28B	0.5238	0.2607	0.2499	0.116*	
H28C	0.5084	0.3464	0.3106	0.116*	
C28A	0.3630 (3)	0.6246 (6)	0.1206 (3)	0.0613 (16)	
H28D	0.3124	0.6022	0.1119	0.092*	
H28E	0.3939	0.5590	0.1048	0.092*	
H28F	0.3733	0.7022	0.0985	0.092*	
C29	0.6241 (4)	0.4452 (7)	0.2559 (4)	0.086 (2)	
H29A	0.5911	0.5078	0.2714	0.128*	
H29B	0.6114	0.4265	0.2105	0.128*	
H29C	0.6736	0.4767	0.2600	0.128*	
C29A	0.4591 (3)	0.6769 (7)	0.2038 (3)	0.0642 (18)	
H29D	0.4727	0.6863	0.2499	0.096*	
H29E	0.4672	0.7551	0.1814	0.096*	
H29F	0.4884	0.6119	0.1854	0.096*	
C30A	0.3769 (3)	1.1011 (6)	0.3224 (3)	0.0581 (16)	
H30A	0.3928	1.1608	0.3557	0.087*	
H30B	0.3569	1.1452	0.2842	0.087*	
H30C	0.4180	1.0510	0.3104	0.087*	
C30	0.6223 (3)	−0.1376 (6)	0.1835 (3)	0.0584 (16)	
H30D	0.6065	−0.2029	0.1533	0.088*	
H30E	0.5809	−0.0868	0.1940	0.088*	
H30F	0.6436	−0.1749	0.2232	0.088*	
C31A	0.4783 (3)	0.8208 (6)	0.4313 (2)	0.0544 (15)	
H31A	0.4705	0.9091	0.4206	0.065*	
H31B	0.4670	0.8098	0.4771	0.065*	
C31	0.5272 (3)	0.1199 (6)	0.0610 (2)	0.0492 (14)	
H31C	0.5287	0.1464	0.0153	0.059*	
H31D	0.5506	0.0377	0.0641	0.059*	
C32	0.4471 (3)	0.1005 (5)	0.0746 (2)	0.0444 (12)	
C32A	0.5587 (3)	0.7926 (5)	0.4255 (2)	0.0467 (13)	
C33	0.4250 (3)	−0.0370 (6)	0.0623 (2)	0.0551 (15)	
C33A	0.5840 (3)	0.6752 (6)	0.4638 (3)	0.0588 (15)	
C34A	0.6670 (3)	0.6802 (6)	0.4688 (3)	0.0732 (19)	
H34A	0.6883	0.6129	0.4436	0.088*	
H34B	0.6858	0.6750	0.5144	0.088*	
C34	0.3416 (3)	−0.0379 (6)	0.0536 (3)	0.0641 (16)	
H34C	0.3254	−0.0700	0.0105	0.077*	
H34D	0.3200	−0.0884	0.0874	0.077*	
C35A	0.6822 (3)	0.8079 (7)	0.4393 (3)	0.0636 (18)	
H35A	0.7296	0.8446	0.4543	0.076*	
C35	0.3222 (3)	0.1002 (6)	0.0607 (3)	0.0534 (14)	
H35B	0.2734	0.1234	0.0417	0.064*	
C36	0.3349 (3)	0.1308 (7)	0.1342 (3)	0.0657 (17)	
H36A	0.3155	0.2133	0.1442	0.079*	
H36B	0.3119	0.0682	0.1612	0.079*	
C36A	0.6711 (3)	0.7949 (9)	0.3634 (3)	0.085 (2)	
H36C	0.6968	0.7220	0.3472	0.102*	
H36D	0.6869	0.8700	0.3409	0.102*	
C37A	0.5852 (3)	0.7777 (8)	0.3559 (3)	0.076 (2)	
H37A	0.5633	0.8412	0.3267	0.091*	
H37B	0.5727	0.6949	0.3384	0.091*	
C37	0.4186 (3)	0.1280 (7)	0.1457 (2)	0.0642 (18)	
H37C	0.4338	0.0619	0.1766	0.077*	
H37D	0.4370	0.2083	0.1626	0.077*	
C38	0.3871 (3)	0.1672 (5)	0.0291 (2)	0.0490 (13)	
C38A	0.6147 (3)	0.8865 (6)	0.4576 (3)	0.0592 (15)	
C39A	0.6112 (4)	1.0181 (7)	0.4278 (4)	0.100 (2)	
H39A	0.6155	1.0126	0.3810	0.150*	
H39B	0.6507	1.0683	0.4469	0.150*	
H39C	0.5650	1.0565	0.4369	0.150*	
C39	0.3850 (3)	0.3113 (5)	0.0368 (3)	0.0627 (16)	
H39D	0.3469	0.3454	0.0075	0.094*	
H39E	0.4315	0.3462	0.0261	0.094*	
H39F	0.3752	0.3323	0.0815	0.094*	
C40	0.3947 (3)	0.1367 (6)	−0.0433 (2)	0.0608 (16)	
H40A	0.3566	0.1786	−0.0693	0.091*	
H40B	0.3907	0.0472	−0.0497	0.091*	
H40C	0.4418	0.1649	−0.0565	0.091*	
C40A	0.6072 (4)	0.8998 (8)	0.5330 (3)	0.089 (2)	
H40D	0.6095	0.8179	0.5531	0.134*	
H40E	0.5609	0.9386	0.5412	0.134*	
H40F	0.6466	0.9512	0.5514	0.134*	
C25	0.9098 (4)	0.1968 (12)	0.3206 (7)	0.198 (5)	
H25A	0.9260	0.1973	0.2763	0.297*	
H25B	0.9516	0.1897	0.3512	0.297*	
H25C	0.8840	0.2738	0.3287	0.297*	
C24	0.8491 (5)	0.0934 (13)	0.4060 (5)	0.172 (4)	
H24A	0.8949	0.0815	0.4309	0.258*	
H24B	0.8163	0.0252	0.4150	0.258*	
H24C	0.8273	0.1718	0.4185	0.258*	
C23A	0.1126 (6)	0.9961 (11)	0.1625 (7)	0.191 (5)	
H23D	0.1422	1.0317	0.1294	0.287*	
H23E	0.1211	1.0415	0.2032	0.287*	
H23F	0.0616	1.0020	0.1483	0.287*	
C24A	0.0731 (5)	0.8508 (15)	0.2237 (6)	0.195 (5)	
H24D	0.0251	0.8606	0.2025	0.292*	
H24E	0.0802	0.9129	0.2578	0.292*	
H24F	0.0775	0.7680	0.2427	0.292*	
C25A	0.1163 (5)	0.7756 (13)	0.1255 (6)	0.229 (6)	
H25D	0.1512	0.7805	0.0917	0.343*	
H25E	0.0676	0.7880	0.1062	0.343*	
H25F	0.1194	0.6941	0.1460	0.343*	
C23	0.8891 (5)	−0.0331 (10)	0.3281 (6)	0.184 (5)	
H23A	0.8971	−0.0583	0.2836	0.276*	
H23B	0.8528	−0.0870	0.3460	0.276*	
H23C	0.9343	−0.0399	0.3543	0.276*	

### Atomic displacement parameters (Å^2^)

**Table:**

	*U*^11^	*U*^22^	*U*^33^	*U*^12^	*U*^13^	*U*^23^
S1	0.0456 (8)	0.0595 (10)	0.0511 (8)	0.0041 (8)	−0.0035 (6)	0.0106 (8)
S1A	0.0441 (8)	0.0642 (10)	0.0454 (7)	0.0038 (8)	−0.0027 (6)	0.0080 (8)
O1	0.061 (2)	0.069 (3)	0.067 (3)	0.017 (2)	0.013 (2)	0.021 (2)
O1A	0.060 (2)	0.067 (3)	0.067 (3)	0.007 (2)	0.0190 (19)	0.011 (2)
O2	0.0339 (18)	0.063 (2)	0.0397 (19)	0.0036 (17)	−0.0045 (14)	0.0054 (18)
O2A	0.0355 (19)	0.066 (3)	0.0376 (19)	−0.0005 (18)	−0.0038 (14)	0.0018 (18)
O3	0.046 (2)	0.079 (3)	0.064 (2)	−0.008 (2)	0.0015 (17)	0.018 (2)
O3A	0.049 (2)	0.089 (3)	0.059 (2)	0.007 (2)	0.0063 (17)	0.021 (2)
O4	0.069 (3)	0.063 (3)	0.076 (3)	0.014 (2)	−0.005 (2)	0.006 (2)
O4A	0.080 (3)	0.057 (3)	0.061 (2)	0.013 (2)	−0.011 (2)	0.007 (2)
O5	0.077 (3)	0.058 (3)	0.080 (3)	0.016 (2)	0.009 (2)	0.015 (2)
O5A	0.089 (3)	0.088 (3)	0.076 (3)	0.001 (3)	−0.010 (2)	0.026 (3)
C1	0.038 (3)	0.047 (3)	0.050 (3)	0.003 (3)	−0.008 (2)	0.004 (3)
C1A	0.044 (3)	0.054 (4)	0.035 (3)	−0.002 (3)	0.000 (2)	0.004 (3)
C2A	0.038 (3)	0.063 (4)	0.054 (3)	−0.006 (3)	−0.005 (3)	0.002 (3)
C2	0.038 (3)	0.059 (4)	0.040 (3)	0.001 (3)	0.004 (2)	0.006 (3)
C3A	0.041 (3)	0.062 (4)	0.059 (3)	0.009 (3)	0.011 (2)	0.002 (3)
C3	0.043 (3)	0.060 (4)	0.055 (3)	0.000 (3)	0.002 (2)	−0.001 (3)
C4A	0.034 (3)	0.071 (4)	0.057 (3)	−0.004 (3)	0.005 (3)	0.011 (3)
C4	0.055 (3)	0.053 (4)	0.051 (3)	0.012 (3)	0.008 (3)	0.005 (3)
C5A	0.043 (3)	0.070 (5)	0.043 (3)	−0.001 (3)	0.003 (2)	0.003 (3)
C5	0.050 (3)	0.061 (4)	0.038 (3)	−0.003 (3)	0.003 (2)	0.007 (3)
C6	0.037 (3)	0.060 (4)	0.034 (3)	−0.006 (3)	−0.002 (2)	0.002 (3)
C6A	0.042 (3)	0.060 (4)	0.030 (3)	0.001 (3)	−0.001 (2)	−0.006 (3)
C7	0.040 (3)	0.054 (4)	0.039 (3)	−0.006 (3)	−0.006 (2)	−0.005 (3)
C7A	0.036 (3)	0.068 (4)	0.038 (3)	0.004 (3)	−0.002 (2)	−0.004 (3)
C8	0.040 (3)	0.050 (3)	0.043 (3)	−0.003 (3)	−0.007 (2)	−0.004 (3)
C8A	0.035 (3)	0.055 (4)	0.037 (3)	0.001 (3)	0.000 (2)	−0.003 (3)
C9A	0.037 (3)	0.057 (4)	0.053 (3)	0.008 (3)	−0.006 (2)	−0.009 (3)
C9	0.043 (3)	0.056 (4)	0.064 (4)	0.007 (3)	−0.005 (3)	−0.010 (3)
C10A	0.041 (3)	0.058 (4)	0.059 (3)	−0.001 (3)	−0.010 (3)	−0.016 (3)
C10	0.050 (4)	0.066 (4)	0.068 (4)	0.004 (3)	−0.021 (3)	−0.006 (4)
C11A	0.045 (3)	0.053 (4)	0.061 (3)	0.002 (3)	−0.013 (3)	−0.018 (3)
C11	0.048 (3)	0.063 (4)	0.063 (3)	0.005 (3)	−0.013 (3)	−0.016 (3)
C12A	0.043 (3)	0.038 (3)	0.050 (3)	0.001 (3)	−0.005 (2)	−0.003 (3)
C12	0.040 (3)	0.048 (3)	0.057 (3)	−0.003 (3)	−0.003 (2)	−0.002 (3)
C13A	0.031 (3)	0.059 (4)	0.037 (3)	0.004 (3)	−0.004 (2)	0.003 (3)
C13	0.037 (3)	0.045 (3)	0.049 (3)	−0.002 (3)	−0.006 (2)	−0.004 (3)
C14	0.046 (3)	0.074 (4)	0.058 (4)	−0.006 (3)	0.008 (3)	0.007 (3)
C14A	0.041 (3)	0.069 (4)	0.059 (4)	−0.001 (3)	0.011 (3)	0.010 (3)
C15A	0.069 (4)	0.106 (6)	0.099 (5)	0.004 (4)	0.042 (4)	0.008 (5)
C15	0.069 (4)	0.092 (5)	0.082 (5)	−0.016 (4)	0.020 (3)	0.016 (4)
C16	0.072 (4)	0.110 (6)	0.049 (3)	0.011 (4)	−0.003 (3)	0.022 (4)
C16A	0.068 (4)	0.114 (6)	0.052 (4)	−0.018 (4)	0.013 (3)	0.017 (4)
C17	0.071 (4)	0.105 (6)	0.074 (4)	0.016 (4)	0.039 (3)	0.026 (4)
C17A	0.070 (4)	0.092 (6)	0.095 (5)	−0.022 (4)	0.010 (4)	0.012 (4)
C18A	0.047 (4)	0.077 (5)	0.129 (6)	0.014 (4)	0.019 (4)	0.047 (5)
C18	0.088 (5)	0.064 (5)	0.069 (4)	0.013 (4)	0.012 (4)	0.012 (4)
C19	0.123 (7)	0.108 (7)	0.220 (11)	0.063 (6)	0.087 (7)	0.078 (8)
C19A	0.110 (7)	0.196 (13)	0.297 (16)	0.058 (8)	0.069 (8)	0.172 (13)
C20A	0.347 (19)	0.207 (15)	0.131 (10)	0.137 (15)	−0.022 (11)	0.076 (10)
C20	0.244 (12)	0.133 (9)	0.081 (6)	0.052 (8)	0.026 (7)	0.034 (6)
C21A	0.083 (6)	0.110 (8)	0.38 (2)	0.002 (6)	0.066 (9)	0.100 (10)
C21	0.289 (16)	0.034 (5)	0.37 (2)	−0.009 (7)	−0.187 (15)	0.035 (8)
C22A	0.056 (4)	0.099 (5)	0.116 (5)	0.031 (4)	−0.046 (3)	−0.041 (4)
C22	0.056 (4)	0.093 (5)	0.133 (5)	0.012 (4)	−0.053 (4)	−0.051 (5)
C26A	0.047 (3)	0.046 (3)	0.052 (3)	−0.001 (3)	0.001 (2)	0.001 (3)
C26	0.051 (4)	0.051 (4)	0.070 (4)	0.003 (3)	−0.007 (3)	−0.014 (3)
C27	0.078 (4)	0.095 (6)	0.090 (5)	0.023 (4)	−0.006 (4)	−0.046 (4)
C27A	0.088 (5)	0.051 (4)	0.091 (5)	0.003 (4)	0.016 (4)	0.002 (4)
C28	0.053 (4)	0.095 (6)	0.085 (5)	0.006 (4)	0.016 (3)	−0.027 (4)
C28A	0.069 (4)	0.066 (4)	0.048 (3)	0.002 (3)	−0.003 (3)	−0.016 (3)
C29	0.095 (5)	0.060 (5)	0.102 (6)	−0.001 (4)	0.013 (4)	−0.013 (4)
C29A	0.048 (3)	0.082 (5)	0.063 (4)	0.002 (3)	0.003 (3)	−0.011 (3)
C30A	0.046 (3)	0.075 (4)	0.054 (3)	−0.005 (3)	0.011 (3)	−0.015 (3)
C30	0.054 (4)	0.073 (4)	0.049 (3)	−0.016 (3)	0.006 (3)	−0.020 (3)
C31A	0.051 (3)	0.073 (4)	0.038 (3)	0.021 (3)	−0.005 (2)	−0.002 (3)
C31	0.044 (3)	0.063 (4)	0.040 (3)	0.004 (3)	−0.005 (2)	0.004 (3)
C32	0.044 (3)	0.057 (3)	0.031 (2)	0.004 (3)	−0.004 (2)	0.007 (2)
C32A	0.041 (3)	0.062 (3)	0.037 (3)	0.006 (3)	0.000 (2)	0.001 (3)
C33	0.063 (4)	0.063 (4)	0.039 (3)	0.012 (3)	0.003 (2)	0.016 (3)
C33A	0.058 (4)	0.070 (4)	0.048 (3)	0.014 (3)	−0.002 (3)	0.003 (3)
C34A	0.054 (4)	0.092 (5)	0.072 (4)	0.029 (3)	−0.010 (3)	−0.001 (4)
C34	0.055 (3)	0.063 (4)	0.074 (4)	−0.006 (3)	0.002 (3)	0.016 (3)
C35A	0.038 (3)	0.091 (5)	0.061 (4)	−0.004 (3)	−0.006 (3)	−0.010 (4)
C35	0.043 (3)	0.062 (4)	0.054 (3)	−0.001 (3)	−0.005 (2)	0.006 (3)
C36	0.050 (3)	0.097 (5)	0.051 (3)	0.009 (3)	0.009 (3)	−0.003 (3)
C36A	0.055 (4)	0.150 (7)	0.051 (4)	−0.002 (4)	0.007 (3)	−0.010 (4)
C37A	0.058 (4)	0.130 (7)	0.038 (3)	0.008 (4)	0.004 (3)	−0.002 (3)
C37	0.059 (4)	0.098 (5)	0.036 (3)	−0.006 (3)	0.006 (2)	−0.001 (3)
C38	0.042 (3)	0.057 (3)	0.047 (3)	0.007 (3)	−0.007 (2)	0.007 (3)
C38A	0.054 (3)	0.072 (4)	0.052 (3)	0.001 (3)	−0.004 (3)	0.000 (3)
C39A	0.091 (5)	0.086 (6)	0.123 (6)	−0.008 (5)	−0.003 (5)	0.013 (5)
C39	0.057 (4)	0.057 (4)	0.074 (4)	0.009 (3)	−0.001 (3)	0.005 (3)
C40	0.074 (4)	0.066 (4)	0.041 (3)	0.012 (3)	−0.013 (3)	0.004 (3)
C40A	0.094 (5)	0.109 (6)	0.062 (4)	0.008 (5)	−0.018 (3)	−0.024 (4)
C25	0.073 (5)	0.180 (9)	0.330 (12)	−0.055 (6)	−0.090 (7)	0.082 (10)
C24	0.128 (7)	0.255 (12)	0.124 (6)	0.031 (7)	−0.066 (5)	0.009 (7)
C23A	0.162 (8)	0.126 (7)	0.267 (11)	0.027 (7)	−0.154 (8)	0.014 (7)
C24A	0.068 (5)	0.297 (13)	0.215 (10)	0.005 (7)	−0.023 (5)	0.004 (9)
C25A	0.136 (7)	0.253 (10)	0.279 (11)	0.093 (8)	−0.158 (7)	−0.173 (10)
C23	0.149 (8)	0.118 (6)	0.267 (10)	0.052 (6)	−0.158 (7)	−0.060 (7)

### Geometric parameters (Å, °)

**Table:**

S1—O4	1.404 (4)	C21—H21D	0.9600
S1—O3	1.424 (3)	C21—H21E	0.9600
S1—O2	1.608 (4)	C21—H21F	0.9600
S1—C31	1.780 (5)	C22A—C25A	1.386 (11)
S1A—O3A	1.425 (3)	C22A—C23A	1.413 (13)
S1A—O4A	1.428 (4)	C22A—C24A	1.542 (11)
S1A—O2A	1.605 (4)	C22—C25	1.374 (12)
S1A—C31A	1.780 (5)	C22—C23	1.448 (12)
O1—C1	1.374 (7)	C22—C24	1.609 (13)
O1—H1A	0.8200	C26A—C29A	1.519 (7)
O1A—C1A	1.379 (7)	C26A—C27A	1.536 (9)
O1A—H1AA	0.8200	C26A—C28A	1.537 (7)
O2—C13	1.431 (5)	C26—C29	1.499 (9)
O2A—C13A	1.440 (5)	C26—C28	1.518 (8)
O5—C33	1.198 (6)	C26—C27	1.543 (8)
O5A—C33A	1.190 (7)	C27—H27A	0.9600
C1—C2	1.393 (7)	C27—H27B	0.9600
C1—C6	1.404 (7)	C27—H27C	0.9600
C1A—C6A	1.383 (7)	C27A—H27D	0.9600
C1A—C2A	1.410 (7)	C27A—H27E	0.9600
C2A—C3A	1.380 (8)	C27A—H27F	0.9600
C2A—C14A	1.546 (8)	C28—H28A	0.9600
C2—C3	1.394 (8)	C28—H28B	0.9600
C2—C14	1.526 (7)	C28—H28C	0.9600
C3A—C4A	1.387 (8)	C28A—H28D	0.9600
C3A—H3AB	0.9300	C28A—H28E	0.9600
C3—C4	1.420 (8)	C28A—H28F	0.9600
C3—H3B	0.9300	C29—H29A	0.9600
C4A—C5A	1.392 (8)	C29—H29B	0.9600
C4A—C18A	1.516 (9)	C29—H29C	0.9600
C4—C5	1.368 (7)	C29A—H29D	0.9600
C4—C18	1.522 (9)	C29A—H29E	0.9600
C5A—C6A	1.382 (8)	C29A—H29F	0.9600
C5A—H5AA	0.9300	C30A—H30A	0.9600
C5—C6	1.390 (8)	C30A—H30B	0.9600
C5—H5A	0.9300	C30A—H30C	0.9600
C6—C7	1.538 (7)	C30—H30D	0.9600
C6A—C7A	1.515 (7)	C30—H30E	0.9600
C7—C30	1.525 (7)	C30—H30F	0.9600
C7—C8	1.533 (7)	C31A—C32A	1.505 (7)
C7—H7A	0.9800	C31A—H31A	0.9700
C7A—C30A	1.511 (7)	C31A—H31B	0.9700
C7A—C8A	1.545 (7)	C31—C32	1.511 (7)
C7A—H7AA	0.9800	C31—H31C	0.9700
C8—C13	1.382 (7)	C31—H31D	0.9700
C8—C9	1.388 (6)	C32—C33	1.529 (8)
C8A—C13A	1.383 (7)	C32—C38	1.562 (6)
C8A—C9A	1.392 (6)	C32—C37	1.586 (7)
C9A—C10A	1.391 (7)	C32A—C37A	1.524 (7)
C9A—H9AA	0.9300	C32A—C33A	1.525 (8)
C9—C10	1.362 (8)	C32A—C38A	1.544 (8)
C9—H9A	0.9300	C33—C34	1.519 (7)
C10A—C11A	1.354 (7)	C33A—C34A	1.510 (8)
C10A—C22A	1.533 (7)	C34A—C35A	1.511 (9)
C10—C11	1.403 (8)	C34A—H34A	0.9700
C10—C22	1.543 (8)	C34A—H34B	0.9700
C11A—C12A	1.407 (6)	C34—C35	1.514 (8)
C11A—H11A	0.9300	C34—H34C	0.9700
C11—C12	1.403 (6)	C34—H34D	0.9700
C11—H11B	0.9300	C35A—C38A	1.546 (8)
C12A—C13A	1.408 (7)	C35A—C36A	1.549 (8)
C12A—C26A	1.552 (7)	C35A—H35A	0.9800
C12—C13	1.404 (7)	C35—C36	1.532 (7)
C12—C26	1.547 (8)	C35—C38	1.546 (7)
C14—C15	1.533 (9)	C35—H35B	0.9800
C14—C17	1.549 (9)	C36—C37	1.530 (7)
C14—C16	1.549 (7)	C36—H36A	0.9700
C14A—C17A	1.493 (9)	C36—H36B	0.9700
C14A—C16A	1.533 (8)	C36A—C37A	1.573 (7)
C14A—C15A	1.537 (9)	C36A—H36C	0.9700
C15A—H15A	0.9600	C36A—H36D	0.9700
C15A—H15B	0.9600	C37A—H37A	0.9700
C15A—H15C	0.9600	C37A—H37B	0.9700
C15—H15D	0.9600	C37—H37C	0.9700
C15—H15E	0.9600	C37—H37D	0.9700
C15—H15F	0.9600	C38—C40	1.518 (7)
C16—H16A	0.9600	C38—C39	1.536 (8)
C16—H16B	0.9600	C38A—C39A	1.520 (10)
C16—H16C	0.9600	C38A—C40A	1.549 (8)
C16A—H16D	0.9600	C39A—H39A	0.9600
C16A—H16E	0.9600	C39A—H39B	0.9600
C16A—H16F	0.9600	C39A—H39C	0.9600
C17—H17A	0.9600	C39—H39D	0.9600
C17—H17B	0.9600	C39—H39E	0.9600
C17—H17C	0.9600	C39—H39F	0.9600
C17A—H17D	0.9600	C40—H40A	0.9600
C17A—H17E	0.9600	C40—H40B	0.9600
C17A—H17F	0.9600	C40—H40C	0.9600
C18A—C19A	1.376 (9)	C40A—H40D	0.9600
C18A—C21A	1.413 (10)	C40A—H40E	0.9600
C18A—C20A	1.626 (11)	C40A—H40F	0.9600
C18—C21	1.493 (10)	C25—H25A	0.9600
C18—C19	1.515 (9)	C25—H25B	0.9600
C18—C20	1.566 (10)	C25—H25C	0.9600
C19—H19A	0.9600	C24—H24A	0.9600
C19—H19B	0.9600	C24—H24B	0.9600
C19—H19C	0.9600	C24—H24C	0.9600
C19A—H19D	0.9600	C23A—H23D	0.9600
C19A—H19E	0.9600	C23A—H23E	0.9600
C19A—H19F	0.9600	C23A—H23F	0.9600
C20A—H20A	0.9600	C24A—H24D	0.9600
C20A—H20B	0.9600	C24A—H24E	0.9600
C20A—H20C	0.9600	C24A—H24F	0.9600
C20—H20D	0.9600	C25A—H25D	0.9600
C20—H20E	0.9600	C25A—H25E	0.9600
C20—H20F	0.9600	C25A—H25F	0.9600
C21A—H21A	0.9600	C23—H23A	0.9600
C21A—H21B	0.9600	C23—H23B	0.9600
C21A—H21C	0.9600	C23—H23C	0.9600
			
O4—S1—O3	118.6 (3)	C29A—C26A—C28A	105.3 (4)
O4—S1—O2	108.7 (2)	C27A—C26A—C28A	106.3 (5)
O3—S1—O2	108.3 (2)	C29A—C26A—C12A	113.4 (5)
O4—S1—C31	109.7 (2)	C27A—C26A—C12A	110.4 (4)
O3—S1—C31	108.8 (3)	C28A—C26A—C12A	110.7 (4)
O2—S1—C31	101.4 (2)	C29—C26—C28	110.4 (5)
O3A—S1A—O4A	118.5 (3)	C29—C26—C27	108.6 (6)
O3A—S1A—O2A	108.8 (2)	C28—C26—C27	105.9 (5)
O4A—S1A—O2A	107.9 (2)	C29—C26—C12	110.4 (5)
O3A—S1A—C31A	109.6 (3)	C28—C26—C12	111.3 (5)
O4A—S1A—C31A	109.9 (2)	C27—C26—C12	110.2 (5)
O2A—S1A—C31A	100.6 (2)	C26—C27—H27A	109.5
C1—O1—H1A	109.5	C26—C27—H27B	109.5
C1A—O1A—H1AA	109.5	H27A—C27—H27B	109.5
C13—O2—S1	121.5 (3)	C26—C27—H27C	109.5
C13A—O2A—S1A	118.7 (3)	H27A—C27—H27C	109.5
O1—C1—C2	117.2 (5)	H27B—C27—H27C	109.5
O1—C1—C6	121.2 (5)	C26A—C27A—H27D	109.5
C2—C1—C6	121.5 (5)	C26A—C27A—H27E	109.5
O1A—C1A—C6A	121.0 (5)	H27D—C27A—H27E	109.5
O1A—C1A—C2A	116.6 (5)	C26A—C27A—H27F	109.5
C6A—C1A—C2A	122.4 (5)	H27D—C27A—H27F	109.5
C3A—C2A—C1A	116.1 (5)	H27E—C27A—H27F	109.5
C3A—C2A—C14A	121.3 (5)	C26—C28—H28A	109.5
C1A—C2A—C14A	122.6 (5)	C26—C28—H28B	109.5
C1—C2—C3	117.4 (5)	H28A—C28—H28B	109.5
C1—C2—C14	122.2 (5)	C26—C28—H28C	109.5
C3—C2—C14	120.3 (5)	H28A—C28—H28C	109.5
C2A—C3A—C4A	124.7 (5)	H28B—C28—H28C	109.5
C2A—C3A—H3AB	117.7	C26A—C28A—H28D	109.5
C4A—C3A—H3AB	117.7	C26A—C28A—H28E	109.5
C2—C3—C4	122.6 (5)	H28D—C28A—H28E	109.5
C2—C3—H3B	118.7	C26A—C28A—H28F	109.5
C4—C3—H3B	118.7	H28D—C28A—H28F	109.5
C3A—C4A—C5A	115.6 (6)	H28E—C28A—H28F	109.5
C3A—C4A—C18A	122.7 (6)	C26—C29—H29A	109.5
C5A—C4A—C18A	121.7 (5)	C26—C29—H29B	109.5
C5—C4—C3	117.0 (5)	H29A—C29—H29B	109.5
C5—C4—C18	122.4 (5)	C26—C29—H29C	109.5
C3—C4—C18	120.6 (5)	H29A—C29—H29C	109.5
C6A—C5A—C4A	123.7 (5)	H29B—C29—H29C	109.5
C6A—C5A—H5AA	118.2	C26A—C29A—H29D	109.5
C4A—C5A—H5AA	118.2	C26A—C29A—H29E	109.5
C4—C5—C6	122.9 (5)	H29D—C29A—H29E	109.5
C4—C5—H5A	118.5	C26A—C29A—H29F	109.5
C6—C5—H5A	118.5	H29D—C29A—H29F	109.5
C5—C6—C1	118.4 (5)	H29E—C29A—H29F	109.5
C5—C6—C7	121.2 (5)	C7A—C30A—H30A	109.5
C1—C6—C7	120.4 (5)	C7A—C30A—H30B	109.5
C5A—C6A—C1A	117.4 (5)	H30A—C30A—H30B	109.5
C5A—C6A—C7A	122.2 (5)	C7A—C30A—H30C	109.5
C1A—C6A—C7A	120.4 (5)	H30A—C30A—H30C	109.5
C30—C7—C8	108.2 (4)	H30B—C30A—H30C	109.5
C30—C7—C6	114.5 (5)	C7—C30—H30D	109.5
C8—C7—C6	113.0 (4)	C7—C30—H30E	109.5
C30—C7—H7A	106.9	H30D—C30—H30E	109.5
C8—C7—H7A	106.9	C7—C30—H30F	109.5
C6—C7—H7A	106.9	H30D—C30—H30F	109.5
C30A—C7A—C6A	114.0 (5)	H30E—C30—H30F	109.5
C30A—C7A—C8A	108.7 (4)	C32A—C31A—S1A	117.9 (4)
C6A—C7A—C8A	112.1 (4)	C32A—C31A—H31A	107.8
C30A—C7A—H7AA	107.2	S1A—C31A—H31A	107.8
C6A—C7A—H7AA	107.2	C32A—C31A—H31B	107.8
C8A—C7A—H7AA	107.2	S1A—C31A—H31B	107.8
C13—C8—C9	118.1 (5)	H31A—C31A—H31B	107.2
C13—C8—C7	122.1 (4)	C32—C31—S1	121.7 (4)
C9—C8—C7	119.1 (5)	C32—C31—H31C	106.9
C13A—C8A—C9A	117.4 (5)	S1—C31—H31C	106.9
C13A—C8A—C7A	122.4 (4)	C32—C31—H31D	106.9
C9A—C8A—C7A	120.1 (5)	S1—C31—H31D	106.9
C10A—C9A—C8A	121.7 (5)	H31C—C31—H31D	106.7
C10A—C9A—H9AA	119.1	C31—C32—C33	110.4 (4)
C8A—C9A—H9AA	119.1	C31—C32—C38	118.9 (4)
C10—C9—C8	122.7 (5)	C33—C32—C38	99.5 (4)
C10—C9—H9A	118.6	C31—C32—C37	120.3 (4)
C8—C9—H9A	118.6	C33—C32—C37	103.2 (4)
C11A—C10A—C9A	117.5 (4)	C38—C32—C37	101.6 (4)
C11A—C10A—C22A	123.5 (5)	C31A—C32A—C37A	116.7 (4)
C9A—C10A—C22A	118.9 (5)	C31A—C32A—C33A	113.1 (5)
C9—C10—C11	117.3 (5)	C37A—C32A—C33A	106.5 (5)
C9—C10—C22	121.1 (6)	C31A—C32A—C38A	117.5 (4)
C11—C10—C22	121.5 (6)	C37A—C32A—C38A	102.9 (4)
C10A—C11A—C12A	125.5 (5)	C33A—C32A—C38A	97.8 (4)
C10A—C11A—H11A	117.2	O5—C33—C34	127.8 (6)
C12A—C11A—H11A	117.2	O5—C33—C32	126.0 (5)
C10—C11—C12	123.5 (5)	C34—C33—C32	106.1 (5)
C10—C11—H11B	118.2	O5A—C33A—C34A	126.0 (6)
C12—C11—H11B	118.2	O5A—C33A—C32A	127.5 (5)
C11A—C12A—C13A	113.5 (5)	C34A—C33A—C32A	106.3 (5)
C11A—C12A—C26A	119.6 (4)	C33A—C34A—C35A	102.0 (5)
C13A—C12A—C26A	126.9 (4)	C33A—C34A—H34A	111.4
C11—C12—C13	115.1 (5)	C35A—C34A—H34A	111.4
C11—C12—C26	119.0 (5)	C33A—C34A—H34B	111.4
C13—C12—C26	125.9 (4)	C35A—C34A—H34B	111.4
C8A—C13A—C12A	124.0 (4)	H34A—C34A—H34B	109.2
C8A—C13A—O2A	115.7 (4)	C35—C34—C33	102.7 (5)
C12A—C13A—O2A	120.0 (4)	C35—C34—H34C	111.2
C8—C13—C12	123.0 (4)	C33—C34—H34C	111.2
C8—C13—O2	117.3 (4)	C35—C34—H34D	111.2
C12—C13—O2	119.5 (4)	C33—C34—H34D	111.2
C2—C14—C15	109.4 (5)	H34C—C34—H34D	109.1
C2—C14—C17	112.1 (5)	C34A—C35A—C38A	103.0 (5)
C15—C14—C17	108.6 (5)	C34A—C35A—C36A	107.3 (6)
C2—C14—C16	109.3 (4)	C38A—C35A—C36A	102.8 (5)
C15—C14—C16	111.1 (6)	C34A—C35A—H35A	114.2
C17—C14—C16	106.3 (5)	C38A—C35A—H35A	114.2
C17A—C14A—C16A	110.4 (6)	C36A—C35A—H35A	114.2
C17A—C14A—C15A	107.9 (5)	C34—C35—C36	105.8 (5)
C16A—C14A—C15A	106.6 (5)	C34—C35—C38	102.6 (5)
C17A—C14A—C2A	111.0 (5)	C36—C35—C38	103.0 (4)
C16A—C14A—C2A	109.0 (4)	C34—C35—H35B	114.7
C15A—C14A—C2A	111.8 (5)	C36—C35—H35B	114.7
C14A—C15A—H15A	109.5	C38—C35—H35B	114.7
C14A—C15A—H15B	109.5	C37—C36—C35	104.2 (4)
H15A—C15A—H15B	109.5	C37—C36—H36A	110.9
C14A—C15A—H15C	109.5	C35—C36—H36A	110.9
H15A—C15A—H15C	109.5	C37—C36—H36B	110.9
H15B—C15A—H15C	109.5	C35—C36—H36B	110.9
C14—C15—H15D	109.5	H36A—C36—H36B	108.9
C14—C15—H15E	109.5	C35A—C36A—C37A	100.6 (4)
H15D—C15—H15E	109.5	C35A—C36A—H36C	111.7
C14—C15—H15F	109.5	C37A—C36A—H36C	111.7
H15D—C15—H15F	109.5	C35A—C36A—H36D	111.7
H15E—C15—H15F	109.5	C37A—C36A—H36D	111.7
C14—C16—H16A	109.5	H36C—C36A—H36D	109.4
C14—C16—H16B	109.5	C32A—C37A—C36A	105.0 (4)
H16A—C16—H16B	109.5	C32A—C37A—H37A	110.7
C14—C16—H16C	109.5	C36A—C37A—H37A	110.7
H16A—C16—H16C	109.5	C32A—C37A—H37B	110.7
H16B—C16—H16C	109.5	C36A—C37A—H37B	110.7
C14A—C16A—H16D	109.5	H37A—C37A—H37B	108.8
C14A—C16A—H16E	109.5	C36—C37—C32	103.6 (4)
H16D—C16A—H16E	109.5	C36—C37—H37C	111.0
C14A—C16A—H16F	109.5	C32—C37—H37C	111.0
H16D—C16A—H16F	109.5	C36—C37—H37D	111.0
H16E—C16A—H16F	109.5	C32—C37—H37D	111.0
C14—C17—H17A	109.5	H37C—C37—H37D	109.0
C14—C17—H17B	109.5	C40—C38—C39	108.3 (5)
H17A—C17—H17B	109.5	C40—C38—C35	114.4 (5)
C14—C17—H17C	109.5	C39—C38—C35	113.1 (5)
H17A—C17—H17C	109.5	C40—C38—C32	112.2 (4)
H17B—C17—H17C	109.5	C39—C38—C32	114.3 (5)
C14A—C17A—H17D	109.5	C35—C38—C32	94.2 (4)
C14A—C17A—H17E	109.5	C39A—C38A—C32A	114.3 (5)
H17D—C17A—H17E	109.5	C39A—C38A—C35A	114.7 (5)
C14A—C17A—H17F	109.5	C32A—C38A—C35A	93.8 (4)
H17D—C17A—H17F	109.5	C39A—C38A—C40A	107.7 (6)
H17E—C17A—H17F	109.5	C32A—C38A—C40A	112.6 (5)
C19A—C18A—C21A	126.2 (9)	C35A—C38A—C40A	113.4 (5)
C19A—C18A—C4A	115.2 (7)	C38A—C39A—H39A	109.5
C21A—C18A—C4A	115.0 (6)	C38A—C39A—H39B	109.5
C19A—C18A—C20A	91.4 (8)	H39A—C39A—H39B	109.5
C21A—C18A—C20A	93.8 (8)	C38A—C39A—H39C	109.5
C4A—C18A—C20A	104.7 (7)	H39A—C39A—H39C	109.5
C21—C18—C19	105.2 (8)	H39B—C39A—H39C	109.5
C21—C18—C4	107.4 (5)	C38—C39—H39D	109.5
C19—C18—C4	113.7 (6)	C38—C39—H39E	109.5
C21—C18—C20	113.7 (9)	H39D—C39—H39E	109.5
C19—C18—C20	106.4 (7)	C38—C39—H39F	109.5
C4—C18—C20	110.5 (6)	H39D—C39—H39F	109.5
C18—C19—H19A	109.5	H39E—C39—H39F	109.5
C18—C19—H19B	109.5	C38—C40—H40A	109.5
H19A—C19—H19B	109.5	C38—C40—H40B	109.5
C18—C19—H19C	109.5	H40A—C40—H40B	109.5
H19A—C19—H19C	109.5	C38—C40—H40C	109.5
H19B—C19—H19C	109.5	H40A—C40—H40C	109.5
C18A—C19A—H19D	109.5	H40B—C40—H40C	109.5
C18A—C19A—H19E	109.5	C38A—C40A—H40D	109.5
H19D—C19A—H19E	109.5	C38A—C40A—H40E	109.5
C18A—C19A—H19F	109.5	H40D—C40A—H40E	109.5
H19D—C19A—H19F	109.5	C38A—C40A—H40F	109.5
H19E—C19A—H19F	109.5	H40D—C40A—H40F	109.5
C18A—C20A—H20A	109.5	H40E—C40A—H40F	109.5
C18A—C20A—H20B	109.5	C22—C25—H25A	109.5
H20A—C20A—H20B	109.5	C22—C25—H25B	109.5
C18A—C20A—H20C	109.5	H25A—C25—H25B	109.5
H20A—C20A—H20C	109.5	C22—C25—H25C	109.5
H20B—C20A—H20C	109.5	H25A—C25—H25C	109.5
C18—C20—H20D	109.5	H25B—C25—H25C	109.5
C18—C20—H20E	109.5	C22—C24—H24A	109.5
H20D—C20—H20E	109.5	C22—C24—H24B	109.5
C18—C20—H20F	109.5	H24A—C24—H24B	109.5
H20D—C20—H20F	109.5	C22—C24—H24C	109.5
H20E—C20—H20F	109.5	H24A—C24—H24C	109.5
C18A—C21A—H21A	109.5	H24B—C24—H24C	109.5
C18A—C21A—H21B	109.5	C22A—C23A—H23D	109.5
H21A—C21A—H21B	109.5	C22A—C23A—H23E	109.5
C18A—C21A—H21C	109.5	H23D—C23A—H23E	109.5
H21A—C21A—H21C	109.5	C22A—C23A—H23F	109.5
H21B—C21A—H21C	109.5	H23D—C23A—H23F	109.5
C18—C21—H21D	109.5	H23E—C23A—H23F	109.5
C18—C21—H21E	109.5	C22A—C24A—H24D	109.5
H21D—C21—H21E	109.5	C22A—C24A—H24E	109.5
C18—C21—H21F	109.5	H24D—C24A—H24E	109.5
H21D—C21—H21F	109.5	C22A—C24A—H24F	109.5
H21E—C21—H21F	109.5	H24D—C24A—H24F	109.5
C25A—C22A—C23A	122.9 (9)	H24E—C24A—H24F	109.5
C25A—C22A—C10A	112.6 (6)	C22A—C25A—H25D	109.5
C23A—C22A—C10A	113.6 (7)	C22A—C25A—H25E	109.5
C25A—C22A—C24A	104.8 (10)	H25D—C25A—H25E	109.5
C23A—C22A—C24A	92.0 (9)	C22A—C25A—H25F	109.5
C10A—C22A—C24A	107.0 (6)	H25D—C25A—H25F	109.5
C25—C22—C23	122.4 (8)	H25E—C25A—H25F	109.5
C25—C22—C10	111.4 (7)	C22—C23—H23A	109.5
C23—C22—C10	112.6 (6)	C22—C23—H23B	109.5
C25—C22—C24	105.2 (9)	H23A—C23—H23B	109.5
C23—C22—C24	93.3 (9)	C22—C23—H23C	109.5
C10—C22—C24	109.3 (6)	H23A—C23—H23C	109.5
C29A—C26A—C27A	110.4 (5)	H23B—C23—H23C	109.5
			
O4—S1—O2—C13	−108.5 (4)	C3A—C4A—C18A—C20A	−126.3 (8)
O3—S1—O2—C13	21.5 (4)	C5A—C4A—C18A—C20A	53.6 (9)
C31—S1—O2—C13	135.9 (4)	C5—C4—C18—C21	77.5 (10)
O3A—S1A—O2A—C13A	−29.8 (4)	C3—C4—C18—C21	−99.1 (9)
O4A—S1A—O2A—C13A	99.9 (4)	C5—C4—C18—C19	−166.5 (7)
C31A—S1A—O2A—C13A	−144.9 (4)	C3—C4—C18—C19	16.8 (9)
O1A—C1A—C2A—C3A	−178.7 (4)	C5—C4—C18—C20	−47.0 (9)
C6A—C1A—C2A—C3A	4.3 (7)	C3—C4—C18—C20	136.4 (7)
O1A—C1A—C2A—C14A	0.4 (7)	C11A—C10A—C22A—C25A	6.6 (13)
C6A—C1A—C2A—C14A	−176.6 (5)	C9A—C10A—C22A—C25A	−175.3 (10)
O1—C1—C2—C3	179.1 (4)	C11A—C10A—C22A—C23A	−138.8 (10)
C6—C1—C2—C3	−1.0 (7)	C9A—C10A—C22A—C23A	39.3 (12)
O1—C1—C2—C14	−2.2 (7)	C11A—C10A—C22A—C24A	121.3 (9)
C6—C1—C2—C14	177.7 (5)	C9A—C10A—C22A—C24A	−60.6 (10)
C1A—C2A—C3A—C4A	−2.1 (8)	C9—C10—C22—C25	115.3 (10)
C14A—C2A—C3A—C4A	178.8 (5)	C11—C10—C22—C25	−68.4 (11)
C1—C2—C3—C4	−1.5 (8)	C9—C10—C22—C23	−26.7 (12)
C14—C2—C3—C4	179.8 (5)	C11—C10—C22—C23	149.6 (9)
C2A—C3A—C4A—C5A	−0.5 (8)	C9—C10—C22—C24	−128.9 (8)
C2A—C3A—C4A—C18A	179.3 (6)	C11—C10—C22—C24	47.4 (10)
C2—C3—C4—C5	3.7 (8)	C11A—C12A—C26A—C29A	147.1 (5)
C2—C3—C4—C18	−179.5 (5)	C13A—C12A—C26A—C29A	−32.4 (7)
C3A—C4A—C5A—C6A	1.3 (8)	C11A—C12A—C26A—C27A	−88.4 (6)
C18A—C4A—C5A—C6A	−178.6 (5)	C13A—C12A—C26A—C27A	92.2 (6)
C3—C4—C5—C6	−3.6 (8)	C11A—C12A—C26A—C28A	29.0 (7)
C18—C4—C5—C6	179.6 (5)	C13A—C12A—C26A—C28A	−150.4 (5)
C4—C5—C6—C1	1.3 (8)	C11—C12—C26—C29	99.1 (6)
C4—C5—C6—C7	−179.8 (5)	C13—C12—C26—C29	−80.9 (7)
O1—C1—C6—C5	−179.0 (4)	C11—C12—C26—C28	−138.1 (6)
C2—C1—C6—C5	1.1 (7)	C13—C12—C26—C28	42.0 (8)
O1—C1—C6—C7	2.1 (7)	C11—C12—C26—C27	−20.9 (8)
C2—C1—C6—C7	−177.8 (4)	C13—C12—C26—C27	159.1 (6)
C4A—C5A—C6A—C1A	0.8 (8)	O3A—S1A—C31A—C32A	158.8 (4)
C4A—C5A—C6A—C7A	−179.5 (5)	O4A—S1A—C31A—C32A	26.9 (5)
O1A—C1A—C6A—C5A	179.5 (4)	O2A—S1A—C31A—C32A	−86.7 (4)
C2A—C1A—C6A—C5A	−3.6 (7)	O4—S1—C31—C32	−47.6 (5)
O1A—C1A—C6A—C7A	−0.3 (7)	O3—S1—C31—C32	−178.8 (4)
C2A—C1A—C6A—C7A	176.6 (4)	O2—S1—C31—C32	67.2 (4)
C5—C6—C7—C30	−25.0 (6)	S1—C31—C32—C33	−144.4 (4)
C1—C6—C7—C30	153.9 (5)	S1—C31—C32—C38	101.6 (5)
C5—C6—C7—C8	99.5 (6)	S1—C31—C32—C37	−24.5 (7)
C1—C6—C7—C8	−81.6 (6)	S1A—C31A—C32A—C37A	45.6 (7)
C5A—C6A—C7A—C30A	25.3 (6)	S1A—C31A—C32A—C33A	−78.5 (5)
C1A—C6A—C7A—C30A	−154.9 (5)	S1A—C31A—C32A—C38A	168.6 (4)
C5A—C6A—C7A—C8A	−98.7 (6)	C31—C32—C33—O5	18.7 (7)
C1A—C6A—C7A—C8A	81.1 (6)	C38—C32—C33—O5	144.4 (5)
C30—C7—C8—C13	−69.5 (7)	C37—C32—C33—O5	−111.1 (6)
C6—C7—C8—C13	162.6 (5)	C31—C32—C33—C34	−160.8 (4)
C30—C7—C8—C9	101.0 (6)	C38—C32—C33—C34	−35.0 (5)
C6—C7—C8—C9	−26.9 (7)	C37—C32—C33—C34	69.4 (5)
C30A—C7A—C8A—C13A	70.8 (7)	C31A—C32A—C33A—O5A	10.3 (8)
C6A—C7A—C8A—C13A	−162.2 (5)	C37A—C32A—C33A—O5A	−119.2 (7)
C30A—C7A—C8A—C9A	−106.3 (6)	C38A—C32A—C33A—O5A	134.7 (6)
C6A—C7A—C8A—C9A	20.7 (7)	C31A—C32A—C33A—C34A	−164.8 (4)
C13A—C8A—C9A—C10A	−2.2 (8)	C37A—C32A—C33A—C34A	65.7 (6)
C7A—C8A—C9A—C10A	175.1 (5)	C38A—C32A—C33A—C34A	−40.3 (5)
C13—C8—C9—C10	0.1 (9)	O5A—C33A—C34A—C35A	−169.2 (6)
C7—C8—C9—C10	−170.8 (5)	C32A—C33A—C34A—C35A	5.9 (6)
C8A—C9A—C10A—C11A	−1.1 (9)	O5—C33—C34—C35	−178.7 (5)
C8A—C9A—C10A—C22A	−179.3 (6)	C32—C33—C34—C35	0.8 (5)
C8—C9—C10—C11	3.3 (9)	C33A—C34A—C35A—C38A	31.1 (5)
C8—C9—C10—C22	179.7 (7)	C33A—C34A—C35A—C36A	−77.0 (5)
C9A—C10A—C11A—C12A	0.2 (9)	C33—C34—C35—C36	−73.0 (5)
C22A—C10A—C11A—C12A	178.4 (6)	C33—C34—C35—C38	34.6 (5)
C9—C10—C11—C12	−2.4 (10)	C34—C35—C36—C37	70.8 (6)
C22—C10—C11—C12	−178.8 (7)	C38—C35—C36—C37	−36.5 (6)
C10A—C11A—C12A—C13A	3.6 (8)	C34A—C35A—C36A—C37A	69.9 (7)
C10A—C11A—C12A—C26A	−175.9 (6)	C38A—C35A—C36A—C37A	−38.2 (7)
C10—C11—C12—C13	−1.7 (9)	C31A—C32A—C37A—C36A	161.6 (6)
C10—C11—C12—C26	178.3 (6)	C33A—C32A—C37A—C36A	−71.1 (7)
C9A—C8A—C13A—C12A	6.6 (8)	C38A—C32A—C37A—C36A	31.3 (7)
C7A—C8A—C13A—C12A	−170.6 (5)	C35A—C36A—C37A—C32A	4.1 (8)
C9A—C8A—C13A—O2A	−179.2 (5)	C35—C36—C37—C32	2.2 (7)
C7A—C8A—C13A—O2A	3.6 (7)	C31—C32—C37—C36	165.8 (5)
C11A—C12A—C13A—C8A	−7.1 (8)	C33—C32—C37—C36	−70.8 (6)
C26A—C12A—C13A—C8A	172.3 (5)	C38—C32—C37—C36	32.1 (6)
C11A—C12A—C13A—O2A	178.9 (5)	C34—C35—C38—C40	61.7 (5)
C26A—C12A—C13A—O2A	−1.6 (8)	C36—C35—C38—C40	171.4 (5)
S1A—O2A—C13A—C8A	87.8 (5)	C34—C35—C38—C39	−173.7 (5)
S1A—O2A—C13A—C12A	−97.8 (5)	C36—C35—C38—C39	−63.9 (6)
C9—C8—C13—C12	−4.6 (8)	C34—C35—C38—C32	−55.0 (5)
C7—C8—C13—C12	165.9 (5)	C36—C35—C38—C32	54.7 (5)
C9—C8—C13—O2	−179.7 (5)	C31—C32—C38—C40	54.8 (7)
C7—C8—C13—O2	−9.1 (8)	C33—C32—C38—C40	−64.9 (5)
C11—C12—C13—C8	5.3 (8)	C37—C32—C38—C40	−170.6 (5)
C26—C12—C13—C8	−174.7 (5)	C31—C32—C38—C39	−69.0 (6)
C11—C12—C13—O2	−179.8 (5)	C33—C32—C38—C39	171.3 (5)
C26—C12—C13—O2	0.2 (8)	C37—C32—C38—C39	65.6 (6)
S1—O2—C13—C8	−81.8 (6)	C31—C32—C38—C35	173.3 (5)
S1—O2—C13—C12	103.0 (5)	C33—C32—C38—C35	53.6 (4)
C1—C2—C14—C15	62.8 (7)	C37—C32—C38—C35	−52.1 (5)
C3—C2—C14—C15	−118.5 (6)	C31A—C32A—C38A—C39A	−63.3 (7)
C1—C2—C14—C17	−176.6 (5)	C37A—C32A—C38A—C39A	66.4 (6)
C3—C2—C14—C17	2.0 (7)	C33A—C32A—C38A—C39A	175.5 (5)
C1—C2—C14—C16	−59.0 (7)	C31A—C32A—C38A—C35A	177.4 (5)
C3—C2—C14—C16	119.6 (6)	C37A—C32A—C38A—C35A	−52.9 (5)
C3A—C2A—C14A—C17A	118.2 (6)	C33A—C32A—C38A—C35A	56.2 (5)
C1A—C2A—C14A—C17A	−60.9 (7)	C31A—C32A—C38A—C40A	60.0 (7)
C3A—C2A—C14A—C16A	−120.0 (6)	C37A—C32A—C38A—C40A	−170.2 (5)
C1A—C2A—C14A—C16A	61.0 (7)	C33A—C32A—C38A—C40A	−61.2 (6)
C3A—C2A—C14A—C15A	−2.4 (8)	C34A—C35A—C38A—C39A	−174.1 (5)
C1A—C2A—C14A—C15A	178.6 (5)	C36A—C35A—C38A—C39A	−62.7 (7)
C3A—C4A—C18A—C19A	−27.6 (11)	C34A—C35A—C38A—C32A	−55.1 (5)
C5A—C4A—C18A—C19A	152.3 (9)	C36A—C35A—C38A—C32A	56.3 (6)
C3A—C4A—C18A—C21A	132.3 (9)	C34A—C35A—C38A—C40A	61.6 (6)
C5A—C4A—C18A—C21A	−47.8 (11)	C36A—C35A—C38A—C40A	173.0 (6)

### Hydrogen-bond geometry (Å, °)

**Table:**

*D*—H···*A*	*D*—H	H···*A*	*D*···*A*	*D*—H···*A*
O1—H1A···O3	0.82	2.23	2.974 (6)	152.
O1A—H1AA···O3A	0.82	2.20	2.957 (6)	153.

## References

[bb1] Bruker (1998). *SAINT-Plus*, *SMART* and *SADABS* Bruker AXS Inc., Madison, Wisconsin, USA.

[bb2] Flack, H. D. (1983). *Acta Cryst.* A**39**, 876–881.

[bb3] Sheldrick, G. M. (2008). *Acta Cryst.* A**64**, 112–122. 10.1107/S0108767307043930 18156677

[bb4] Spek, A. L. (2009). *Acta Cryst.* D**65**, 148–155. 10.1107/S090744490804362X PMC2631630 19171970

[bb5] Wu, J., Yu, T.-L., Chen, C.-T. & Lin, C.-C. (2006). *Coord. Chem. Rev.* **250**, 602–626.

